# Tibial Spine Fracture in an Adolescent Male After Minor Injury: A Case Report

**DOI:** 10.5811/cpcem.2022.9.57228

**Published:** 2022-10-27

**Authors:** Alberto Nunez, Shayna Sleight, Zara Khan, Barbara Blasko, Tommy Y. Kim

**Affiliations:** * University of California, Riverside School of Medicine, Riverside, California; † HCA Healthcare, Riverside Community Hospital, Department of Emergency Medicine, Riverside, California

**Keywords:** tibial spine, fracture, avulsion

## Abstract

**Case Presentation:**

A 13-year-old male presented with right knee pain and swelling from a basketball injury. The right knee exam demonstrated minimal swelling, decreased range of motion secondary to pain, and generalized tenderness. A radiograph of the right knee revealed a tibial spine fracture.

**Discussion:**

Tibial spine fractures are avulsion fractures of the spine of the tibia at the insertion site of the anterior cruciate ligament. The incidence of avulsion fractures is higher in adolescents because the region of the apophyseal growth plate between the soft-tissue attachment site and the body of the bone is weaker in that age group. Tibial spine avulsion fractures are relatively uncommon and occur annually in approximately three per 100,000 children.

## CASE PRESENTATION

**Image 1:**
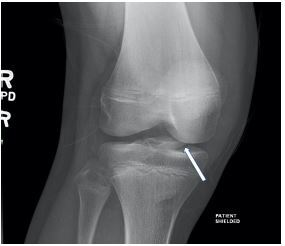
Anteroposterior view of right tibial spine avulsion fracture in a teenage boy.

**Image 2:**
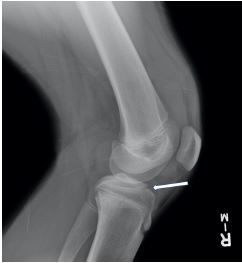
Lateral view of right tibial spine avulsion fracture showing anterior displacement.

A 13-year-old male was brought to the emergency department with right knee pain. Prior to the visit, he had been playing basketball and reported 7/10 pain upon jumping. He was unsure whether he had twisted his knee when landing. The right knee exam demonstrated minimal swelling, decreased range of motion, and generalized tenderness. The remaining examination of the right lower extremity, including the hip and ankle, was normal. A radiograph of the right knee revealed a tibial spine fracture (Images 1 and 2). The patient was placed in a knee immobilizer and analgesics were recommended. He was instructed to be non-weight bearing with crutches and was to follow up with orthopedics within the following two weeks.

## DISCUSSION

Tibial spine fractures are avulsion fractures of the spine of the tibia at the insertion site of the anterior cruciate ligament. The incidence of avulsion fractures is higher in adolescence because the region of the apophyseal growth plate between the soft tissue attachment site and the body of the bone is weaker in this age group.^1^ When soft tissues such as tendons and ligaments attached to the bone develop a force capable of overcoming the stress capacity of the attachment, this force can tear off a portion of the bone leading to an avulsion fracture. Common lower-extremity avulsion fractures in adolescents occur at the ischial tuberosity, anterior superior iliac spine, and the anterior inferior iliac spine. Other less common lower-extremity avulsion fractures occur at the tibial tuberosity, calcaneus, and the greater and lesser trochanters.^2^

Tibial spine avulsion fractures are relatively uncommon and occur annually in approximately three per 100,000 children. The incidence is generally higher in boys compared to girls.^3^ Tibial spine fractures typically occur during an athletic activity or trauma. The mechanism leading to injury is a combination of hyperextension and internal rotation at the knee. Associated soft tissue injuries such as meniscal or ligament tears and/or entrapment of the meniscus occurs in up to 59% of patients, which may influence the management plan of the fracture between immobilization vs surgery.^4^

Standard anteroposterior and lateral knee radiographs have conventionally been used to diagnose tibial spine fractures. Computed tomography can provide further detail about the fracture (such as the extent of displacement), and magnetic resonance imaging can allow assessment of the menisci, cartilage, and ligaments. Classifications of tibial spine fractures are based on severity and displacement: Type I fractures are non-displaced; Type II fractures are displaced anteriorly with an intact posterior hinge; Type III fractures are completely displaced; and Type IV fractures are those that are completely displaced and comminuted.^3^ Generally, non-operative management with immobilization is indicated for isolated Type I and mild Type II fractures.^3^ Operative management is generally indicated for Types III and IV fractures, which leads to better functional outcomes and patient satisfaction.^3^

**CPC-EM Capsule**

What do we already know about this clinical entity?

*Avulsion fractures are common in the adolescent years. Tibial spine fractures are relatively uncommon compared to other lower extremity avulsion fractures*.

What makes this presentation of disease reportable?

*Avulsion fracture of the tibial spine occurred in an adolescent after a minor knee injury*.

What is the major learning point?

*Although uncommon, avulsion fractures do occur in adolescents with knee injuries often associated with ligamentous injuries*.

How might this improve emergency medicine practice?

*Practitioners should thoroughly evaluate x-rays for associated avulsion fractures such as tibial spine fractures in any adolescent with a joint injury*.
